# Investing in mental health in Somalia: harnessing community mental health services through task shifting

**DOI:** 10.1017/gmh.2022.4

**Published:** 2022-02-22

**Authors:** Mohamed Ibrahim, Mamunur Rahman Malik, Zeynab Noor

**Affiliations:** 1 The University of British Columbia, Vancouver, British Columbia, Canada; 2 WHO Somalia, Mogadishu, Somalia; 3 Mental Health Department Somalia Federal Ministry of Health, Mogadishu, Somalia

**Keywords:** Somalia, mental health care, task-shifting/sharing, mhGAP, UHC

## Abstract

**Background:**

The increase of mental health issues globally has been well documented and now reflected in the United Nations' Sustainable Development Goals as a matter of global health significance. At the same time, studies show the mental health situations in conflict and post-conflict settings much higher than the rest of the world, lack the financial, health services and human resource capacity to address the challenges.

**Methods:**

The study used a descriptive literature review and collected data from public domain, mostly mental health data from WHO's Global Health Observatory. Since there is no primary database for Somalia's public health research, the bibliographic databases used for mental health in this study included Medline, PubMed, CINAHL, PsycINFO, and Google Scholar.

**Results:**

The review of the mental health literature shows one of the biggest casualties of the civil war was loss of essential human resources in healthcare as most either fled the country or were part of the victims of the war.

**Conclusion:**

In an attempt to address the human resource gap, there are calls to task-shift so that available human resource can be utilized efficiently and effectively. This policy paper discusses the case of Somalia, the impact of decade-long civil conflict on mental health and health services, the significant gap in mental health service delivery and how to strategically and evidently task-shift in closing the mental health gap in service delivery.

## Why mental health matters, especially in conflict-affected settings: a situational analysis of Somalia

### Global perspective

Globally, mental, neurological and substance use disorders affect about 10% of the general population, but in countries affected by humanitarian crises, one in every five people is estimated to suffer from some form of mental disorder (Marquez, 2018; Ryan *et al*., 2021). More than 75% of people with mental, neurological and substance use conditions in low- and middle-income countries (LMIC), especially in conflict and post-conflict settings, do not have access to effective mental health services (Ryan *et al*., 2021). The lack of treatment increases the burden of illness in terms of mortality, morbidity, stigma and discrimination (Ryan *et al*., 2021). Evidence shows that mental illness is a leading cause of disability globally (Chisholm *et al*., 2016).

Economically, mental disorders lead to reduced productivity, unemployment, loss of wages and resultant poverty, which affects the financial well-being at the individual level and eventually at the national and global levels (Marquez, 2018; Ryan *et al*., 2021, Chisholm *et al*., 2016). Depression and anxiety alone cost the global economy about a trillion US dollars a year, while the total financial cost of mental illness is expected to exceed US$ 6 trillion by 2030 (WHO, 2016). LMIC bear more than 50% of these costs (Chisholm *et al*., 2016). Despite the enormous financial consequences of mental health, tackling mental illness is still less of a priority in LMIC, with most such countries allocating <2% of their health budget to mental health services (Marquez, 2018, Vigo *et al*., 2016).

**Table:** 

‘COVID-19 has interrupted essential mental health services around the world just when they're needed most,’ and calling on world leaders to ‘move fast and decisively to invest more in life-saving mental health programmes – during the pandemic and beyond’ as ‘good mental health is absolutely fundamental to overall health and well-being’. Dr Tedros Adhanom Ghebreyesus, 2020 Director General, World Health Organization

In 2016, the World Bank (Kovacevic, 2021), International Monetary Fund (IMF), World Health Organization (WHO) and global partners reiterated that returns on investment on treatments for mental health far outweigh the cost. A 2016 cost–benefit analysis of investing in mental health treatment for depression and anxiety in 36 low/middle- and high-income countries for the 15 years from 2016 to 2030 concluded that there would be a fourfold return on investment for every dollar spent (Chisholm *et al*., 2016).

### The Somali context

Three decades of conflict and violence in Somalia have led to economic marginalization and social exclusion of young people in Somalia, who make up about 70% of the population, as well as other vulnerable people such as women and children and internally displaced people. These factors drive poverty and the resultant health inequity in the country. In Somalia, it is estimated that the prevalence of mental health illness is much higher than global estimates with one in every three people affected by some form of mental illness (Abdillahi *et al*., 2020). Years of conflict and the effects of climate shocks have contributed to widespread psychosocial trauma and social deprivation in Somalia with devastating consequences on people's mental health (Abdillahi *et al*., 2020). Although the burden of disease in Somalia (Institute for Health Metrics and Evaluation, 2020*a*) is dominated by communicable diseases, maternal, neonatal and nutritional disorders which represent 68% of disability-adjusted life years, mental disorders constitute a significant burden of disease as it tops at the 13th most disabling condition as calculated by years lived with disabilities (Institute for Health Metrics and Evaluation, 2020*b*).

The conflict and instability in Somalia, climate shocks and the recent coronavirus disease 2019 (COVID-19) pandemic have delivered a triple blow to the country, substantially increasing the need for mental health and psychosocial support. The country's mental health services are almost non-existent with just 0.5 psychiatric beds/100 000 population compared to 6.4 beds/100 000 in the WHO Eastern Mediterranean Region and 24 beds/100 000 globally (WHO, 2019). With the exception of a few understaffed and poorly resourced psychiatric hospitals, Somalia has no community-based mental health services.

Given the country's current situation with the health workforce, it is unlikely that Somalia will be able to train enough mental health specialists in the foreseeable future. Therefore, it is prudent, realistic and strategic to adopt a task-shifting model (WHO, 2007) in which non-specialists provide mental health services integrated within primary health care using the WHO Mental Health Gap Action Programme (WHO, 2008*a*, 2008*b*).

### Scaling up integrated community mental health services through task-sharing

**Table:** 

The WHO Mental Health Gap Action Programme aims to scale up services for mental, neurological and substance use disorders especially in low- and middle-income countries. The programme asserts that with proper care, psychosocial assistance and medication – even where resources are scarce – tens of millions of people could be treated for depression, schizophrenia and epilepsy and prevented from suicide, and could begin to lead normal lives. (WHO, 2008*a*, 2008*b*).

The health sector in Somalia has embarked on the implementation of a transformative agenda aimed at rebuilding and reorganizing its health system to achieve universal health coverage (UHC) using primary health care services as the entry point. The government is expected to roll out soon an essential package of health services (EPHS) with an integrated service delivery model with primary health care at its heart. The package is a set of cost-effective and evidence-informed interventions to be delivered across all service platforms in the country. This package will bring the health services close to families, ensure a continuum of care across all service delivery platforms and improve access and coverage.

Against the backdrop of the roll out of the EPHS, and with the great demand for mental health services, the treatment gap and unmet needs must be strategically tackled. This can be done through a task-shifting model which strengthens and uses the existing health infrastructure and workforce, and increases community outreach and support by enlisting more community health workers including the newly launched female health workers or *Marwo Caafimad* in the Somali language. The *Marwo Caafimad* programme was jointly developed by the federal ministry of health, UNFPA, WHO and UNICEF in an attempt to address the huge gap in health human response, especially with the understanding of the high rates of maternal and infant mortality in the country (MoH, 2014). The *Marwo Caafimad* programme is seen as cost-effective, culturally appropriate and sustainable interventions to address the huge gap in primary health care in the country and enhancing integrated health service delivery (MoH, 2014). Therefore, the Mental Health & Psychosocial Services (MHPSS) task shifting through community-based integrated health services is consistent with the government's strategies for achieving UHC (2019–2023) and the implementation plan for the EPHS (MoH, 2021).

Although no evidence shows the effectiveness of this new *Marwo Caafimad* model, the existing political and structural support system for integrating community health workers in the healthcare system in Somalia could be an ingredient for success and overcome the systemic challenges often faced by LMIC countries in implementing task-shifting model (Javadi *et al*., 2017)

### COVID-19 and its mental health consequences

A worldwide global survey undertaken by WHO looking into the impact of COVID on mental health services (2020). With a response rate of 67% (130 countries), the result showed that 93% of all the countries reported disruption of one or more of mental health services during the pandemic (WHO, 2020). The mental health services hard hit during the lockdowns were community mental health services, residential care and addiction treatment programmes. While public mental health programmes such as school mental health programmes, older adults, youth services and antenatal and postnatal mental health services were significantly disrupted (2020). These are essential mental health services at the most critical time for the most vulnerable sectors of the society (2020).

Somalia and the rest of the Eastern Mediterranean Region of WHO comprising of 21 countries across Horn of Africa (Somalia and Djibouti), North Africa, Middle East, South Asia (Pakistan and Afghanistan) with a population of nearly 700 million host half of all the 70 million displaced population in the world (Said *et al*., 2021). With high rates of mental health conditions in humanitarian settings, the current global pandemic is making such scenario even worse, where pandemic-related conditions are leading to psychological distress, anxiety and depression. While at the same access to already limited mental health services and supplies disrupted due to the shutdowns especially lifesaving drugs for psychosis, depression and epilepsy (Said *et al*., 2021).

There is a significant dearth of research and publication on the mental health impact of COVID in the context of Somalia however, existing studies from similar humanitarian settings indicates a significant increase of COVID-related mental health conditions. For example, reports showed an increase in suicide and suicide attempts and domestic and gender-based sexual violence among refugees in Lebanon and Jordan (Said *et al*., 2021).

Resource-limited countries such as Somalia are the least likely capable of addressing the serious challenges posed by a pandemic of such magnitude. The healthcare system in Somalia has never developed beyond providing the most basic functions and has negligible capacity to the level of mental health conditions facing its citizens. It is within the current pandemic situation that it becomes more urgent to introduce robust task shifting in mental health and psychosocial support service provision to addressing existing and COVID-19-related challenges.

### WHO's pyramid framework for mental health services

**Fig. 1.**
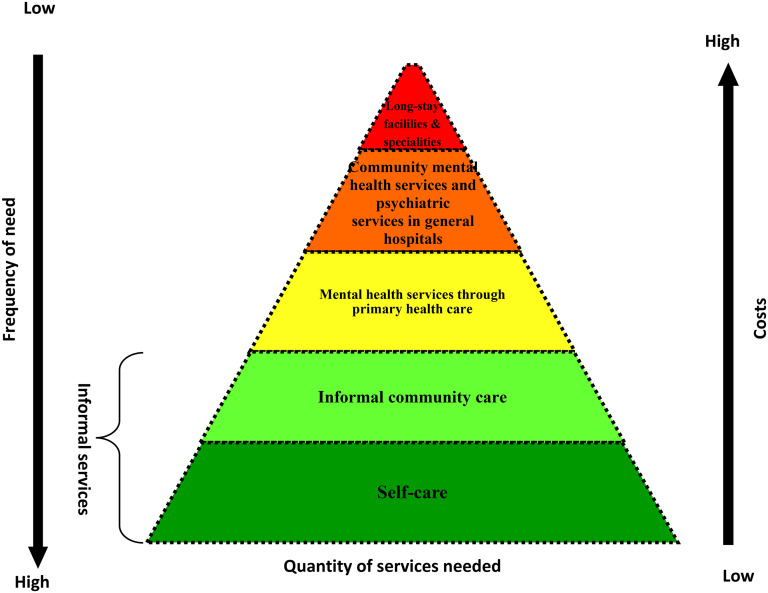
WHO pyramid framework of optimal mix of services (source: WHO MIND project).

WHO has developed a pyramid framework for an optimal mix of services to provide guidance to countries on how to organize mental health services using the community-based approach (Fig. 1). The pyramid framework illustrates that most mental health care can be achieved at the community level through integrated health care services by training non-specialist health professionals (physicians, nurses and midwives) on the WHO Mental Health Gap Action Programme and using community health workers and/or volunteers as a bridge between informal and formal health care services (WHO, 2017).

Using this approach, several countries have successfully integrated mental health services into primary health care services with encouraging results (Table 1).

**Table 1.** Examples of integrated mental health services in primary health care in low- and middle-income settings

Country	Examples of integrated mental health services in primary health care	Key features	Outcome
Uganda	Integrated primary health care for mental health care in Sembabule District (WHO, 2017)	Inclusion of mental health in the basic minimum health package Inclusion of village health teams (volunteers) for awareness, identification and referral Outreach services attached to the regional hospital Training of nurses, midwives and clinical officers at primary health care level Formation of service-user organizations	Uptake of outpatients services Increased patient satisfaction with primary care model Appropriate referral to general hospitals Accessible services at the community level
Belize	Nationwide district mental health care in primary health care (WHO, 2017)	Preventive mental health care, outpatient services, HIV counselling, gender based and sexual violence, crisis management, school health and outreach services	Substantial uptake of mental health services at the community level Decrease in psychiatric hospital admissions and long-term stays
Kerala, India	Integrating primary care for mental health in Thirunvananthapura District (WHO, 2017)	Formation of district mental health programme Training of non-specialists Inclusion and training of *anganwadi* (community health care) workers Formation of service user organizations Community outreach, school mental health promotion	Uptake of services for bipolar disorder, schizophrenia, depression and epilepsy 25 outreach programmes across the district Referral network between primary health care and hospitals
South Africa	Integrated primary care services for mental health in the Ehlanzeni District, Mpumalanga Province (WHO, 2017)	Mental health services integrated in primary health care and managed by nurses with monthly support of psychiatrists	Referral of acute and serious cases to district and regional hospitals 83% coverage of mental health care across both districts by 2007

In addition to the countries in Table 1, Argentina, Belize, Chile, Islamic Republic of Iran and Saudi Arabia have successfully implemented integrated mental health services in primary health care either nationally or regionally with varying degree of success depending on government commitment, funding and capacity (WHO, 2008*a*, 2008*b*). The programmes were able to successfully manage priority conditions as reflected in the Mental Health Gap Action Programme.

### Fostering peace through tackling mental health: the case of Somalia

Somalia's 30-year-old civil strife has severely disrupted social cohesion, broken down social norms and led to widespread psychological suffering. Long-standing conflict undermines trust between individuals, families, communities and their institutions (World Youth Report, 2003). With 70% of Somali's population under the age of 30 years, the vast majority of the population was born and grew up in the midst of conflict. This situation can lead children, young people and adults to normalize and potentially reproduce violence and conflict through retribution, joining armed groups and intimate partner violence (World Youth Report, 2003). Studies of adverse childhood experiences and trauma, such as hunger, violence and neglect, have shown an association with long-term chronic health conditions including mental health and substance use (Dube *et al*., 2003). Therefore, neglecting to address the psychosocial impact of conflict will ultimately undermine peace, health and development.

The WHO country office in Somalia, in partnership with the IOM, UNICEF and the federal government, is currently implementing a pilot project on youth-oriented integrated MHPSS in the context of peace building. The project complements existing primary health care services and peace-building initiatives and tackles an important service delivery gap – MHPSS – that is currently not covered by any humanitarian or developmental programmes in Somalia. As part of this project, WHO and implementing partners are conducting a study on the links between mental health, conflict and peace building in order to provide evidence about the interplay between MHPSS and drivers of conflict in Somalia, and help to inform new evidence-based approaches and interventions that can be implemented as a follow-on to the project. It is being increasingly recognized among professionals working in MHPSS and peace building that interventions aiming to achieve build peace would benefit from closer links with mental health interventions, as both add vital elements to rebuilding social, economic and political structures.

### Task shifting for integrated mental health services

The interventions in the Mental Health Gap Action Programme for priority mental, neurological and substance use conditions and the recommended psychological interventions, if implemented to scale, can fill the gap in treatment and mental health services in Somalia. These services can be delivered by integrating mental health services through, for example, the following task shifting approaches.

- Community outreach level: community and female health workers can be trained on: screening for common mental health illness; health education; psychosocial interventions that can be done at the community level; and referral pathways to primary health care units.
- Primary health care level: frontline primary health care workers (nurses, physicians, midwives, public health officers and social workers) can be trained to recognize and treat mental health illnesses that are treatable at the primary health care level in line with the intervention package in the Mental Health Gap Action Programme.
- General hospital level: referral networks established, mental health inpatient units integrated in selected general hospitals, and nurses, physicians and midwives trained on the Mental Health Gap Action Programme.

Investing in mental health is investment in development and peace building – two vital elements for post-conflict rebuilding of Somalia.
